# Popular Medicine

**Published:** 1902-03-15

**Authors:** 


### Popular Medicine.

In spite of the extreme specialisation of function rendered necessary by our highly complex social system, it is nevertheless a fact that people insist more and more on settling for themselves questions requiring highly technical knowledge. We have long been accustomed to the man in the street giving his opinion on politics, religion, art and literature with an amount of assurance only equalled by his ignorance of the subject commented upon, but it is only of recent years that he has also assumed a dogmatic attitude towards science. Perhaps some of our lay contemporaries are not altogether irresponsible for this, for as a rule the new and marvellous remedies for consumption, the latest ideas on vaccination, and the up-to-date method of capturing the supposed cancer germ, which some confiding friend whispers to us at the dinner table, have been derived from some form of printed matter whose title is generally forgotten. Nevertheless the doctor who happens to forget the name of the special species of mosquito which carries the malarial germ is not regarded with much respect by the young lady who has just read up the subject in a magazine article. It is not only in what we may call the academic side of medicine, but also in the practical treatment of disease that the public is assuming a new attitude. It is true the doctor is still consulted, but he is no longer regarded with a certain amount of awe as one who has acquired some marvellous skill and knowledge not vouchsafed to ordinary mortals. Indeed, the patient usually has not only clearly made up his mind concerning the nature of his malady, but is far more apt to consider the physician wrong, if he disagrees, than to alter his own diagnosis. Further, a great number of people refuse to take medicine unless they know what are the remedies or even the nature of the ingredients which are prescribed, although it is impossible to realise what information is conveyed to any but an expert chemist by such a statement, for example, as that lycetol is di-methyl-piperazine-tartrate. A stockbroker recently stated that his business was much less arduous to-day than 20 years ago, because people, instead of spending hours in his office discussing the merits of certain investments, now settled the whole thing for themselves, and merely wired instructions to buy or sell so many shares at a certain price. The doctor of the future will probably be told on his arrival by his patient what is the ailment, and also what is the only drug which will be of use, and his duty will therefore be simplified to writing an order for it to the chemist. He will therefore have as little to say as the modern stockbroker. It must not, however, be assumed that the doctor will be entirely relieved from responsibility in the future.

If anything goes wrong or the patient does not recover in the number of days or hours which he has settled for himself, his illness will last, then it will not be difficult to guess who will be blamed. Fortunately, however, when there is real danger, things assume quite another aspect, for fear always drives out the confidence of ignorant peoples. There is a great deal of quiet humour in it all, and often it is an interesting source of speculation as to what is in the mind of a person for example who seriously tells one that the mucous membrane of his œsophagus has lost tone. One wonders also what the peritoneum is supposed to be by those of the public who talk so glibly of peritonitis, or how a strong will power is presumed to act to prevent influenza. Speaking of the latter disease, the latest popular idea seems to be that the infection can only be removed from the patient by a course of tonics, but it is not explained how this will act. A young probationer, in answering a question on antiseptics in an examination paper, stated that they acted best when wet, because then the wings of the germs were rendered so heavy that they "could not fly away." One wonders whether the man in the street also pictures the tubercle bacillus as a very diminutive form of house-fly, when he is telling one of the latest methods, supposed to be practised at Brompton Hospital, of "sluicing" the lungs several times a day with a new antiseptic which is not poison to the human being. What also is the popular idea of a lung? Such a treatment is, however, not nearly so marvellous as the operation of taking out the eye, washing it, and putting it back again, which is supposed to be performed every day by our ophthalmic surgeons. There is a folk-song society which is flourishing greatly, folk lore also has its students, why should not folk medicine also be approached as a serious subject?

But let us as scientific people look to ourselves, and remember that our opinions on subjects outside our own little groove may be as absurd as the man in the street's ideas on medicine appear to us.

